# Potential Biomarker Identification by RNA-Seq Analysis in Antibiotic-Related Drug Reaction with Eosinophilia and Systemic Symptoms (DRESS): A Pilot Study

**DOI:** 10.1093/toxsci/kfac062

**Published:** 2022-06-15

**Authors:** Ying Xin Teo, Wei Yann Haw, Andreas Vallejo, Carolann McGuire, Jeongmin Woo, Peter Simon Friedmann, Marta Ewa Polak, Michael Roger Ardern-Jones

**Affiliations:** Clinical Experimental Sciences, Faculty of Medicine, University of Southampton, Southampton SO16 6YD, UK; Department of Dermatology, Southampton General Hospital, University Hospitals Southampton NHS Foundation Trust, Southampton SO16 6YD, UK; Clinical Experimental Sciences, Faculty of Medicine, University of Southampton, Southampton SO16 6YD, UK; Clinical Experimental Sciences, Faculty of Medicine, University of Southampton, Southampton SO16 6YD, UK; Clinical Experimental Sciences, Faculty of Medicine, University of Southampton, Southampton SO16 6YD, UK; Clinical Experimental Sciences, Faculty of Medicine, University of Southampton, Southampton SO16 6YD, UK; Clinical Experimental Sciences, Faculty of Medicine, University of Southampton, Southampton SO16 6YD, UK; Clinical Experimental Sciences, Faculty of Medicine, University of Southampton, Southampton SO16 6YD, UK; Clinical Experimental Sciences, Faculty of Medicine, University of Southampton, Southampton SO16 6YD, UK; Department of Dermatology, Southampton General Hospital, University Hospitals Southampton NHS Foundation Trust, Southampton SO16 6YD, UK

**Keywords:** biomarker, diagnostics, drug allergy, drug reaction with eosinophilia and systemic symptoms (DRESS), immunology

## Abstract

One of the most severe forms of cutaneous adverse drug reactions is “drug reaction with eosinophilia and systemic symptoms” (DRESS), hence subsequent avoidance of the causal drug is imperative. However, attribution of drug culpability in DRESS is challenging and standard skin allergy tests are not recommended due to patient safety reasons. Whilst incidence of DRESS is relatively low, between 1:1000 and 1:10 000 drug exposures, antibiotics are a commoner cause of DRESS and absence of confirmatory diagnostic test can result in unnecessary avoidance of efficacious treatment. We therefore sought to identify potential biomarkers for development of a diagnostic test in antibiotic-associated DRESS. Peripheral blood mononuclear cells from a “discovery” cohort (*n* = 5) challenged to causative antibiotic or control were analyzed for transcriptomic profile. A panel of genes was then tested in a validation cohort (*n* = 6) and compared with tolerant controls and other inflammatory conditions which can clinically mimic DRESS. A scoring system to identify presence of drug hypersensitivity was developed based on gene expression alterations of this panel. The DRESS transcriptomic panel identified antibiotic-DRESS cases in a validation cohort but was not altered in other inflammatory conditions. Machine learning or differential expression selection of a biomarker panel consisting of 6 genes (STAC, GPR183, CD40, CISH, CD4, and CCL8) showed high sensitivity and specificity (100% and 85.7%–100%, respectively) for identification of the culprit drug in these cohorts of antibiotic-associated DRESS. Further work is required to determine whether the same panel can be repeated for larger cohorts, different medications, and other T-cell-mediated drug hypersensitivity reactions.

Drug hypersensitivity caused by T-cell-mediated reactions are clinically distinct in their presentation from IgE-mediated drug allergy reactions and present as a range of different clinical phenotypes (Brockow *et al.*, 2019), including drug reaction with eosinophilia and systemic symptoms (DRESS). DRESS typically presents with a florid skin eruption combined with hallmark systemic features of fever, lymphadenopathy, blood dyscrasias such as eosinophilia, and internal organ involvement (Ardern-Jones and Mockenhaupt, 2019; Brockow *et al.*, 2019; Shiohara *et al.*, 2007). The liver is the most commonly involved among the organs, found in 51%–94.2% of patients; followed by renal involvement, lung, cardiac, and central nervous system (Chen *et al.*, 2010; Hiransuthikul *et al.*, 2016; Kardaun *et al.*, 2013; Martínez-Cabriales *et al.*, 2019). Future lifelong avoidance of the culprit drug is crucial as DRESS can be life-threatening, reported mortality being 2%–6% (Ardern-Jones and Mockenhaupt, 2019; Kardaun *et al.*, 2013; Wu *et al.*, 2018). Confirmation of causality can be difficult if the culprit drug is not clinically obvious.

Skin tests and oral challenge cannot be performed acutely and are generally not recommended because of the risk of reinducing DRESS. Clinical algorithms to assess causality are of value, especially for postmarketing surveillance systems, but their lack of confirmatory testing limits their utility to inform treatment decisions for an individual patient (Sassolas *et al.*, 2010). We and others have demonstrated the diagnostic use of classical immunology tests to measure drug specific T cell activation (Haw *et al.*, 2016; Polak *et al.*, 2013). However, such *in vitro* assays are not widely available due to being labor intensive, complex, and involving radioisotopes. Therefore, there is an unmet need to develop a simple, quick, and robust *in vitro* assay that can be undertaken widely in routine diagnostic laboratories.

We set out to develop an *in vitro* gene transcription signature to identify drug-induced cell activation because reverse transcriptase polymerase chain reaction (RT-PCR)-based assays are already widely employed in clinical laboratories and therefore this approach would be scalable to routine laboratories. To determine the optimal biomarkers for drug T cell activation, we undertook ribonucleic acid-sequencing (RNA-seq) of drug-exposed peripheral blood mononuclear cells (PBMCs) from antibiotic-induced DRESS cases, as these were the cases most frequently referred for further diagnostic work-up in our center. Differential expression from control samples identified candidate genes as markers of drug hypersensitivity, which were further validated against a second cohort, against tolerant controls, and other inflammatory conditions.

## MATERIALS AND METHODS

###  

####  

##### Patients and controls

Eleven antibiotic-associated DRESS patients, as confirmed by RegiSCAR score ≥3 and with positive results on lymphocyte proliferation or enzyme-linked immunosorbent spot (ELISpot) assay testing (Kardaun *et al.*, 2007), were recruited to the study through the Department of Dermatology, University Hospital Southampton NHS Foundation Trust. Causative antibiotics included: cefoxitin, dapsone, teicoplanin, and vancomycin. Ethical approval was obtained from the Health Research Authority (17/NE/0346). Only subjects with no active infections or malignancies and without history of immunosuppression were included. Patients were divided into a “discovery” cohort (*n* = 5) and a “validation” cohort (*n* = 6) (Table 1). Seven comparative tolerant controls were also tested. All testing was undertaken on fresh (not frozen) samples isolated from anticoagulated peripheral blood. The tests were undertaken on average (mean) 370.7 days from rash onset (median: 124 days, IQR 71–347).

**Table 1.** Demographics of Tested Subjects and Comparative T-Cell Assay Results

Cohort	Sex	Age Range (Years)	Phenotype	Drug	RegiSCAR Score	LPA (*C*_max_ SI)	IFN-γ [*C*_max_ –(background + 2× SD)]
Discovery	M	35–40	DRESS	Cefoxitin	3	69.9*a*	254
*n* = 5	M	25–30	DRESS	Cefoxitin	5	63.4*a*	74
	F	75–80	DRESS	Vancomycin	3	7.67*a*	10
	M	45–50	DRESS	Teicoplanin	6	50.4*a*	175
	F	70–75	DRESS	Dapsone	5	18.5*a*	111
Validation	M	20–25	DRESS	Cefoxitin	3	13.7*a*	20
*n* = 6	F	15–20	DRESS	Cefoxitin	3	3.6*a*	21
	M	35–40	DRESS	Cefoxitin	3	2.3*a*	39
	M	70–75	DRESS	Vancomycin	5	2.5*a*	554
	F	40–45	DRESS	Vancomycin	4	18.4*a*	113
	F	80–85	DRESS	Teicoplanin	3	1.3	605
Tolerant controls	F	25–30	Tolerant	Cefoxitin	NA	1.7	Neg
*n* = 7	M	20–25	Tolerant	Cefoxitin	NA	0.7	Neg
	F	80–85	Tolerant	Vancomycin	NA	2.1	Neg
	F	80–85	Tolerant	Vancomycin	NA	0.6	Neg
	M	55–60	Tolerant	Vancomycin	NA	1.4	Neg
	F	65–70	Tolerant	Vancomycin	NA	1.2	Neg
	M	60–65	Tolerant	Teicoplanin	NA	0.8	Neg

*C*_max_, maximal concentration; IFN, interferon; LPA, lymphocyte proliferation assay; NA, not applicable; Neg, negative; RegiSCAR, registry of Severe Cutaneous Adverse Reaction (RegiSCAR score: 2–3 possible case, 4–5 probable case, >5 definite case); SD, standard deviation.

a Positive result (SI > 2).

##### Lymphocyte proliferation and ELISpot test

Lymphocyte proliferation test and IFN-γ ELISpot assay were performed as described previously (Haw *et al.*, 2016; Polak *et al.*, 2013). Each drug was tested to 4 different concentrations, with 4-fold dilutions performed starting from the following highest concentrations: cefoxitin 128.25 μg/ml, dapsone 0.74 μg/ml, teicoplanin 51.28 μg/ml, and vancomycin 434.79 μg/ml.

##### RNA isolation and Purification

PBMCs (7.5 × 10^5^ cells per well, in duplicates) were incubated for 24 h with medium (control) or culprit drug at concentrations with observed highest responses on LPA and ELISpot testing (cefoxitin 32.06 μg/ml, dapsone 0.19 μg/ml, teicoplanin 12.82 μg/ml, and vancomycin 217.40 μg/ml) before RNA harvesting for transcriptomic analysis. Following this, PBMCs were harvested, washed, and suspended in RLT lysing buffer (Qiagen, UK) before storage at −20°C. Each sample was thawed immediately before RNA isolation and whole transcriptome RNA-sequencing. RNA extraction and purification were performed according to manufacturer’s protocol (RNeasy Plus Mini Kit, Qiagen, UK). DNA contamination in the collected RNA was eliminated by use of gDNA Eliminator spin column. RNA quantity and quality checking were performed using the NanoDrop 1000 spectrophotometer (Thermo Fisher Scientific, Waltham, Massachusetts) and Agilent 2100 Bioanalyzer. All samples displayed a 260/280 ratio >1.8 and RNA integrity numbers (RIN) > 7.7. Purified RNA samples were stored at −80°C until use.

##### mRNA-Seq library construction and sequencing

Total RNA samples were subjected to indexed cDNA library construction, using the Illumina TruSeq poly(A) + RNA-Seq library construction, according to the manufacturer’s instructions. For sequencing, all samples were pooled in a single pool and sequenced on 3 lanes, yielding 75-bp paired-end reads, using an Illumina HiSeq 4000 platform (an outsourced service at the Oxford Genomics Centre).

##### Bioinformatics analysis

Quality-controlled reads were aligned to the reference genome GRCh37.EBVB95-8wt.ERCC using the HISAT aligner. Alignments were counted for each gene using the featureCounts package (Liao *et al.*, 2014). Aligned reads were further analyzed in R using the Bioconductor suite of packages. Filtered trimmed mean of M (TMM) values normalized counts per million (cpm) (EdgeR, filtering out genes less than 2 gene counts in at least half of the samples) were used for downstream analyses (Robinson *et al.*, 2010). Determination of differentially expressed genes (DEG) was performed using EdgeR with a nested paired design (Robinson *et al.*, 2010). The expected false discovery rate (FDR) was estimated using the Benjamini-and-Hochberg method. An FDR adjusted *p* ≤ .05 was considered significant.

RNA-seq data were deposited in accordance with MIAME guidelines, in Gene Expression Omnibus (GEO) under accession number GSE160369.

##### Quantitative reverse transcription-PCR

The expression of chosen genes was validated with quantitative PCR using the TaqMan gene expression assays for target genes: *YWHAZ* (Hs01122445_g1), *STAC* (Hs00182385_m1), *CISH* (Hs00367082_g1), *FN1* (Hs01549976_m1), and *CD4* (Hs01058407_m1) (Applied Biosystems, Life Technologies, UK) in PBMCs isolated from whole blood. RNA extraction (RNeasy Plus Mini Kit, Qiagen) and cDNA reverse transcription, including RT-negative control, (High-Capacity cDNA Reverse Transcription Kit, Applied Biosystems; ThermoFisher Scientific UK) were carried out according to the manufacturer’s protocol. qPCR was performed in 384-well plate assay, using Applied Biosystems 7900HT Fast Real-Time PCR System. Gene expression levels were normalized to housekeeping gene expression (*YWHAZ*).

##### TaqMan array card

Customized RT-PCR cards from Applied Biosystems (http://www.appliedbiosystems.com; last accessed June 2021) were used in the quantitative analysis of the 22 selected candidate genes. Eight samples with 2 technical duplicates were tested per card. The 384-well microfluidic card was preloaded with our chosen genes. Each cDNA sample was added to an equal volume of mastermix (TaqMan, Applied Biosystems) and then loaded onto the array card. PCR amplification was performed using a 7900HT Fast Real-time PCR System (Applied Biosystems) following the protocol described by the manufacturer. Relative expression of each gene was normalized to *YWHAZ* as the sole housekeeping gene, and log2-transformed for analysis (RQ = 2^−^^ΔΔ^^*C*^^t^). All data were generated in duplicate for each gene expression per sample.

##### Evaluation of diagnostic performances

Ranking of detected genes for selection of candidate biomarker genes was done using absolute log fold change (FC) cut-off (logFC≥|1.5|) calculated using generalized linear model in EdgeR, combined with minimum expression levels for all donors (minimum cpm ≥ 4, maximum cpm ≥ 100). Random forest analysis was performed using package Ranger in R (importance measure = impurity, number of trees = 500, α = 0.9). Combinatorial panel analysis with top 10 candidate genes identified on random forest algorithm were performed using CombiRoc webtool (Mazzara *et al.*, 2017). Receiver-operating characteristic (ROC) curves were calculated in order to assess the diagnostic power of the gene combination by the area under the curve (AUC) of the ROC curve. Potential biomarkers were considered valuable if sensitivity and specificity were >85%, as well as AUC ≥ 0.8.

##### Comparison with systemic inflammatory conditions

Datasets for 4 systemic conditions: influenza, sepsis, systemic lupus erythematosus, and dermatomyositis were downloaded from publicly available genomic data repositories (GSE114588, GSE60424, GSE112087, and GSE125977). Transcriptome analysis was from PBMC in the influenza and sepsis datasets whilst sequencing was performed on whole blood in the other diseases. FASTQ files for GSE114588 and GSE60424 were aligned using Kallisto (Bray *et al.*, 2016) against the GRCh38 human reference genome followed by differential analysis using Sleuth (Pimentel *et al.*, 2017). Disease describing gene expression signatures were generated by comparing TMM normalized gene expression levels between experimental and control group using EdgeR package (Robinson *et al.*, 2010) (FC ≥ log2 and adjusted *p* value < .05). Raw RNA-seq data for GSE112087 was quantified to gene-level counts using the ARCHS4 pipeline (Lachmann *et al.*, 2018) with similar thresholds as the other datasets. Published values (FC ≥ log2) relating to dermatomyositis subjects from GSE125977 were extracted for comparative analysis. Enrichment analyses performed to published gene sets associated with these 4 inflammatory conditions (influenza 2, sepsis 5, systemic lupus erythematosus 5, and dermatomyositis 1) did not show significant overlap (enrichment scores: 0.27–0.55, FDR < 0.05).

##### Functional enrichment analysis

Gene set enrichment analysis (Mootha *et al.*, 2003; Subramanian *et al.*, 2005) was performed for complete DRESS dataset (11747 transcripts, average were calculated for transcripts associated with the same genes [3 genes]) using curated gene signatures of 4 inflammatory diseases above downloaded from MSigDB (Molecular Signatures Database v7.1) (Supplementary Table 5). Largest collections relating to dermatomyositis from DisGeNET platform (v7.0) (Piñero *et al.*, 2015, 2020) were used in view of no available curated gene sets for this disease on other MSigDB platform (Liberzon *et al.*, 2015; Subramanian *et al.*, 2005). Similarities were examined at cut-off of FDR-adjusted *p* value < .05 and enrichment scores.

##### Scoring classification

Mean values for each biomarker gene was calculated from RT-qPCR data from 6 DRESS subjects tested using the array card and compared against logFC RNAseq data to determine up- and downregulated genes in the identified panel. For every transcript expression which matched this expected change, 1 point was added whilst 1 point was subtracted if direction of change was opposite to that of the identified signature. Log_2_  2^−^^ΔΔ^^*C*^^t^ values for each subject (6 DRESS, 7 tolerant controls) were used in this scoring. No points were added or subtracted if values fell between −0.25 and 0.25.

##### Statistics

Statistical analyses were performed using Prism 8.1 (GraphPad Software) and methods embedded in bioinformatics pipelines (generalized linear model, EdgeR, Benjamini-and-Hochberg FDR-corrected *p* value test). Mann-Whitney *U* test was used for comparison between nonmatched nonparametric samples and Fisher’s exact test for contingency table analysis. Correlations between RNA-seq and qPCR results were performed using Pearson test and linear regression analysis. Data were considered significant at *p* < .05.

## RESULTS

###  

#### Correlation Between Clinical Diagnosis and *In Vitro* Assays

**Figure 1.**
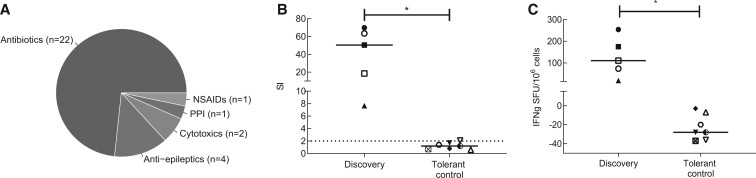
Causative drugs in referred DRESS cases and confirmation of clinically suspected antibiotic by positive T cell assay in DRESS “discovery” cohort. A, Prevalence of causative drug groups in DRESS cases referred to Southampton NHS Foundation Trust between 2017 and 2018. B, Lymphocyte proliferation test (LPA) measured as stimulation index (SI) of proliferation induced by drug versus media control. C, IFN-γ release in drug-induced responses measured by ELIspot in “discovery” cohort subjects (*n* = 5), and control patients tolerant of similar antibiotics (*n* = 7). Each data point represents maximum measured response to tested drug. Horizontal solid lines indicate group median. Horizontal dotted line shows positive result threshold. Mann-Whitney *U* test used for assessing statistical significance, *p value < .05. Abbreviations: DRESS, drug reaction with eosinophilia and systemic symptoms; IFN-γ ELIspot, interferon-gamma enzyme-linked immunosorbent spot; LPA, lymphocyte proliferation assay; NSAID, nonsteroidal anti-inflammatory; PPI, protein pump inhibitor; SFU, spot forming unit.

DRESS was the most common presentation of DHR in Southampton tertiary referral center (53% of diagnosed DHR in 2017–2018) and in our cohort, antibiotics were the dominant causal drugs for this condition (Figure 1A). Five cases of antibiotic-induced DRESS were selected (“discovery” cohort). Causative antibiotics include: cefoxitin, vancomycin, teicoplanin, and dapsone. Cohort characteristics (median age 49 years, IQR: 36–71), are described in Table 1. We confirmed that all identified antibiotic-DRESS cases demonstrated positive *in vitro* responses to stimulation with the culprit antibiotic, whereas no drug-induced responses were detected in tolerant controls (LPA *p* = .0025, IFN-γ *p* = .0025, Mann-Whitney *U* test) (Figs. 1B and 1C).

#### Antibiotic Exposure Induces Transcriptomic Programs Encoding Immune Activation in PBMCs from DRESS Patients

**Figure 2.**
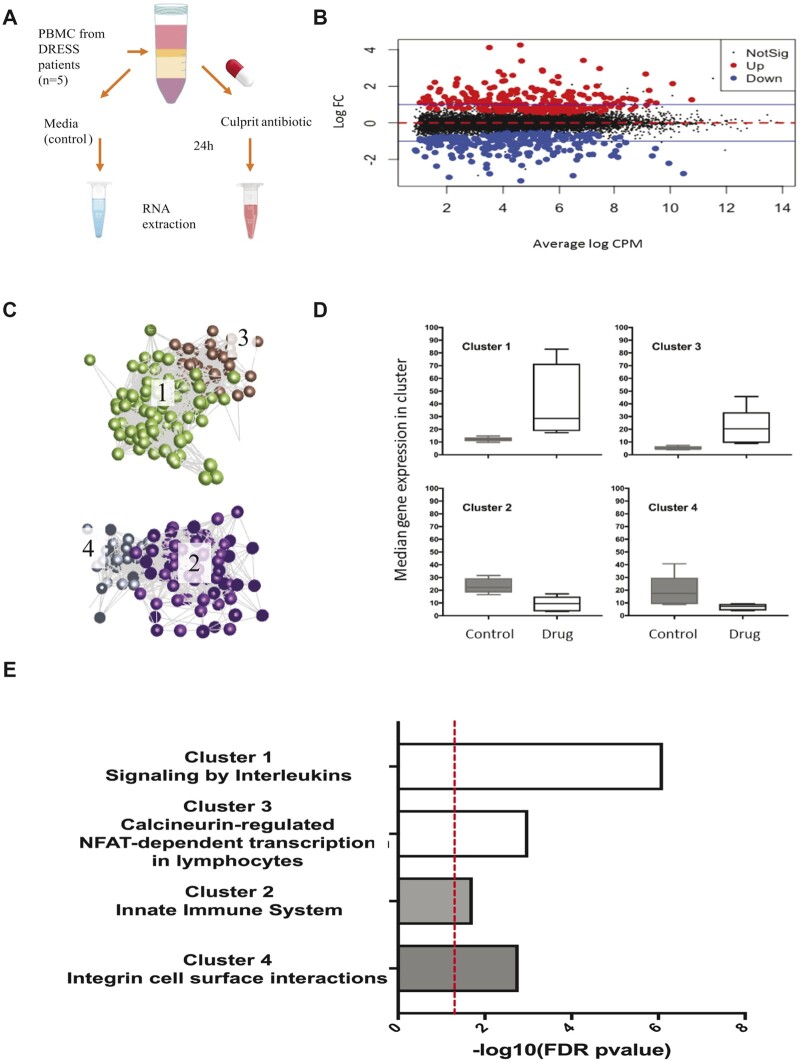
Testing protocol and identification of differentially expressed genes. A, PBMCs were cultured in culture media supplemented or not with culprit drug at the optimized concentration for 24 h before RNA extraction. B, MA plot representation of 267 drug-specific DEGs (149 upregulated, red; 118 downregulated, blue; FDR *p* < .05 blue line depicts a threshold of logFC ≥|1|). C–E, Transcript-to-transcript correlation network analysis of gene expression changes induced by culprit drug in DRESS patients (discovery cohort, *n* = 5). 4 major clusters shown, cluster 1 (green, *n* = 103 genes), cluster 2 (purple, *n* = 76 genes), cluster 3 (brown, *n* = 39 genes), and cluster 4 (gray, *n* = 32 genes). Each node (dot) indicates a transcript, each line defines the Pearson correlation coefficient between a pair of nodes (GraphiaPro, Pearson *r* ≥ 0.85, MCL = 1.7). D, Median gene expression profiles in clusters 1–4 in control (gray) and drug exposed cells (white). Box and whiskers indicate median ± range. E, Key processes identified by gene ontology analysis specific to each cluster (ToppGene, FDR cut-off 0.05, cluster 1: FDR *p* = 7.67 × 10^−7^, cluster 2: FDR *p* = 1.87 × 10^−2^; cluster 3: FDR *p* = 1 × 10^−3^; cluster 4: FDR *p* = 1.65 × 10^−3^). Abbreviations: DEG, differentially expressed gene; FC, fold change; FDR, false discovery rate. A color version of this figure appears in the online version of this article.

To identify transcriptomic biomarkers specific for DRESS induced by antibiotics, discovery cohort PBMCs were cocultured with culprit drug or control *in vitro* for 24 h before isolation of RNA for transcriptome profiling (Figure 2A). This identified 267 drug-specific DEGs (149 up and 118 downregulated; EdgeR, FDR *p* < .05, logFC ≥|1|, Figure 2B). Transcript-to-transcript clustering (GraphiaPro, Pearson *r* ≥ 0.85, MCL = 1.7) identified 4 main clusters (Figure 2C). Clusters 1 and 3, comprising 141 genes in total, were enriched in genes regulating cytokine receptor activity (Cluster 1, FDR *p* = 7.67 × 10^−7^) and T cell activation via NFAT (Cluster 3, FDR *p* = 1 × 10^−3^, Figs. 2D and 2E). In contrast, genes in clusters 2 and 4 were downregulated, and indicated modulation of innate immune system function (Cluster 2, FDR *p* = 1.87 × 10^−2^) and reduced integrin interactions (Cluster 4, FDR *p* = 1.65 × 10^−3^, Figs. 2D and 2E).

#### Identification of Candidate Molecular Biomarkers for DRESS

**Figure 3.**
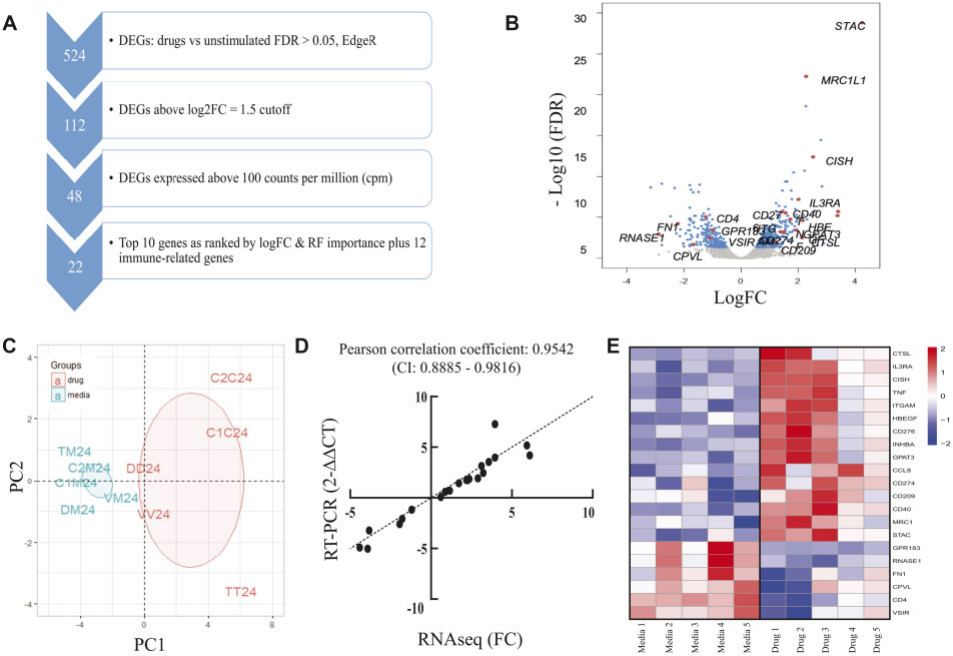
Identification of candidate biomarker genes. A, Selection of candidate biomarkers. Following identification of 524 DEGs by comparison of drug-stimulated and media (unstimulated) in the discovery cohort (EdgeR package, FDR100 were selected). Ten genes with absolute FC ≥ |2| and random forest (RF) importance ≥ 0.05 were selected from the filtered genes and combined with 12 immune-related genes to form the gene panel. B, Volcano plot of genes measured in DRESS discovery cohort, differentiating responses to culprit drug versus media control. Differentially expressed genes (FDR < 0.05) shown in blue (upregulated genes on right, downregulated on left), genes selected indicated in red. C, PCA clustering (first 2 components) comparing signature panel gene expression induced by culprit drug (red) and media (blue) after 24-h culture. D, Comparison of gene changes detected in panel genes using RNAseq and PCR with customized microfluidic array card in a single subject, normalized to YWHAZ gene expression. E, Heatmap depicting changes in expression of selected 22 candidate biomarkers in 5 antibiotic-DRESS patients exposed to culprit drug versus media control. Color indicates the expression change in logFC. Red: upregulated genes; blue: downregulated genes. Abbreviations: DEG, differentially expressed gene; FC, fold change; PC, principal component; PCA, principal component analysis; RF, random forest. A color version of this figure appears in the online version of this article.

To select a panel of candidate biomarkers, DEGs exceeding |logFC| ≥ 1.5 were filtered for the nominal gene expression value (minimum cpm ≥ 4 for all the donors, at least 100 cpm). The resulting 48 candidate biomarkers were evaluated for predictive value using a random forest algorithm in R (package Ranger, α = 0.9, number of trees = 500). The top 10 genes with absolute FC ≥ |2| (up and downregulation) and RF importance ≥ 0.05 (Figs. 3A and 3B) and 12 additional immune-related genes were included in the final candidate biomarker panel (Figure 3A, full list of genes including 2 housekeeping genes in Supplementary Table 1). Unsupervised principal component clustering of the candidate biomarkers confirmed that they efficiently differentiated drug-exposed cells from their media control counterparts (Figure 3C). RNA-seq analysis was validated using RT-qPCR for the top 4 gene transcripts (Supplementary Figure 1) and a customized array card confirming the differential expression profile of all 22 transcripts (*r* = 0.9542 *p* = < .0001, Figure 3D). The differential expression of the candidate biomarker panel (Figure 3E) highlights that although these 22 genes differentiates drug-exposed cells from the control, a degree of heterogeneity existed in expression of specific genes between different subjects.

#### DRESS Biomarkers Are Specific to Drug Hypersensitivity

**Figure 4.**
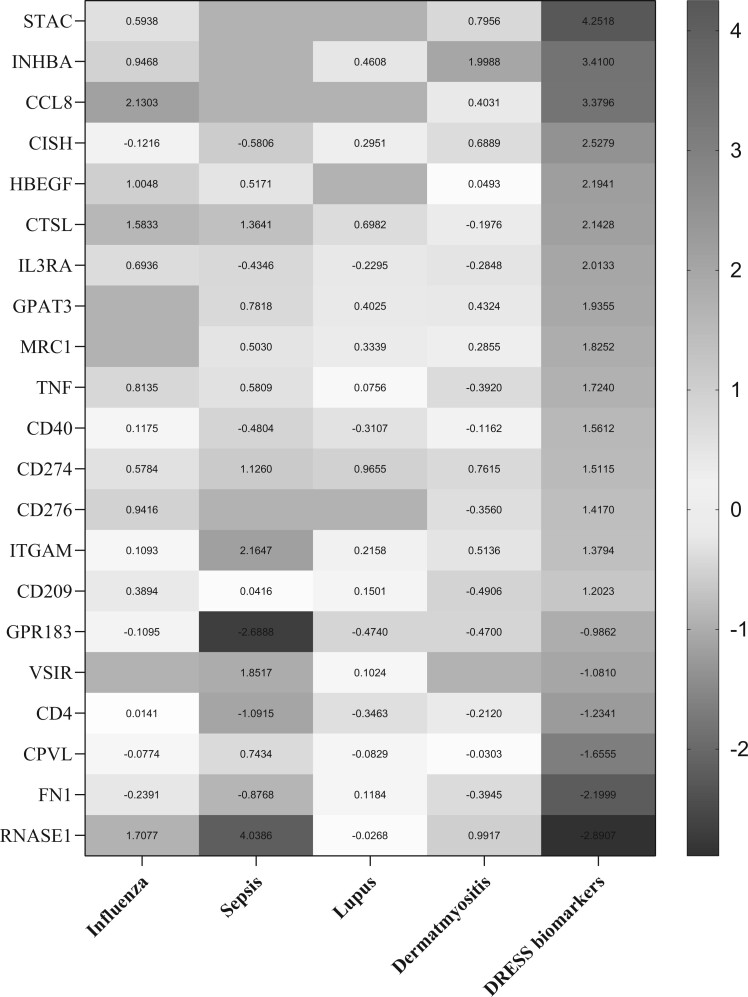
DRESS biomarkers are specific to drug hypersensitivity. Heatmap depicting expression of biomarker gene panel in samples sourced from public data repositories including influenza infection (GSE114588), sepsis (GSE60424), systemic lupus erythematosus (GSE112087), and dermatomyositis (GSE125977). Color indicates the expression change compared with DRESS allergics. Red: upregulated genes. Blue: downregulated genes, gray: not differentially expressed. A color version of this figure appears in the online version of this article.

To determine if the identified biomarker panel was DRESS specific, we undertook a comparative analysis with influenza infection (GSE114588), sepsis (GSE60424), systemic lupus erythematosus (GSE112087), and dermatomyositis (GSE125977). Gene expression in these 4 conditions differed markedly from DRESS (Figure 4) and showed low correlations between DRESS and influenza (0.351), sepsis (−0.179), systemic lupus erythematosus (0.327), and dermatomyositis (0.321) (Pearson correlation coefficient).

#### Validation of DRESS Gene Panel

**Figure 5.**
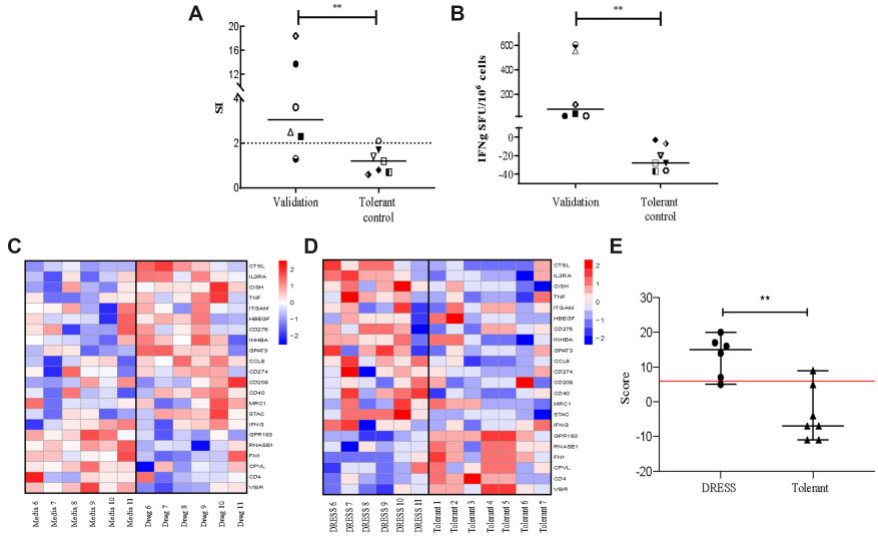
DRESS validation cohort confirms specificity of biomarker panel. A–B, Characteristics of *in vitro* responses to culprit drug in antibiotic-DRESS validation cohort (*n* = 6) and control patients tolerant of similar antibiotics (*n* = 7). A, Lymphocyte proliferation test (LPA) measured as stimulation index (SI) of proliferation induced by drug versus media control. B, IFN-γ release in drug-induced responses measured by ELISpot. Each data point represents maximum measured response to tested drug. Each data point represents maximum measured response to tested drug. Horizontal solid lines indicate group median. Horizontal dotted line shows positive result threshold. Mann-Whitney *U* test used for statistical significance (** = *p* < .01). C, Heatmap depicting changes in expression of selected 22 candidate biomarkers in validation DRESS cohort exposed to culprit drug versus media control. Color indicates the expression change in logFC. Red: upregulated genes; blue: downregulated genes. D, Heatmap depicting changes in expression of selected 22 candidate biomarkers in validation DRESS cohort versus tolerant patients. Color indicates the expression change in logFC. Red: upregulated genes, blue: downregulated genes. E, Box and whisker plot showing cumulative scoring using 22 biomarker genes compared with expected expression alterations based on signature panel. Error bars indicate data range. Horizontal red line indicates threshold score considered positive. (** = *p* < .01, Fisher’s exact test). Abbreviations: IFN-γ ELIspot, interferon-gamma enzyme-linked immunosorbent spot; SFU, spot forming unit. A color version of this figure appears in the online version of this article.

To confirm the candidate molecular biomarker panel, we prospectively identified a “validation cohort” (6 cases of DRESS caused by antibiotics: cefoxitin, vancomycin and teicoplanin) as well as patients tolerant of the same antibiotics (*n* = 7). This group was similar in terms of age, sex, and time to onset (Table 1). Similar to the discovery cohort, positive tests for drug hypersensitivity were demonstrated by T cell functional assays *in vitro* (LPA *p* = .0082, IFN-γ *p* = .0012, Mann-Whitney *U* test) in all DRESS subjects (Figs. 5A and 5B). To validate the gene signature panel, PBMCs from allergics were challenged with culprit medications, and the 22-candidate biomarker panel analyzed. Comparison of culprit drug against media control in DRESS patients (Figure 5C) and between DRESS cohort against tolerant individuals (Figure 5D) showed clearly identifiable differences. In tolerant subjects, the 22 candidate biomarkers tested were only minimally affected following exposure to antibiotics (median change in gene expression relative to *YWHAZ* for each gene 2^−^^ΔΔ^^*C*^^t^ = 1.04, range: 0.68–1.81), confirming the signature was specific for DRESS. As expected, some heterogeneity in the gene expression patterns between individuals was evident in both tolerant controls and allergic individuals.

#### An Algorithm for Analysis of Gene Expression Alterations as a Diagnostic Approach in Antibiotic-DRESS

A point attribution system based on observed changes in each of the transcripts from the 22-gene biomarker panel was developed. Scoring 6 DRESS subjects and 7 tolerant controls showed statistically significant difference (*p* = .0052, Mann-Whitney *U* test) when scored against all 22 genes (Figure 5E, full scores listed in Supplementary Table 2). By setting a threshold score of 6, this novel scoring system was able to correctly stratify almost all cases (5 DRESS, 6 controls) with high sensitivity and specificity (83.3% and 85.7% respectively, *p* = .029, Fisher’s exact test).

#### Machine Learning Identifies Optimal Panel of Biomarkers Differentiating Antibiotic-DRESS Patients From Tolerant Controls

**Figure 6.**
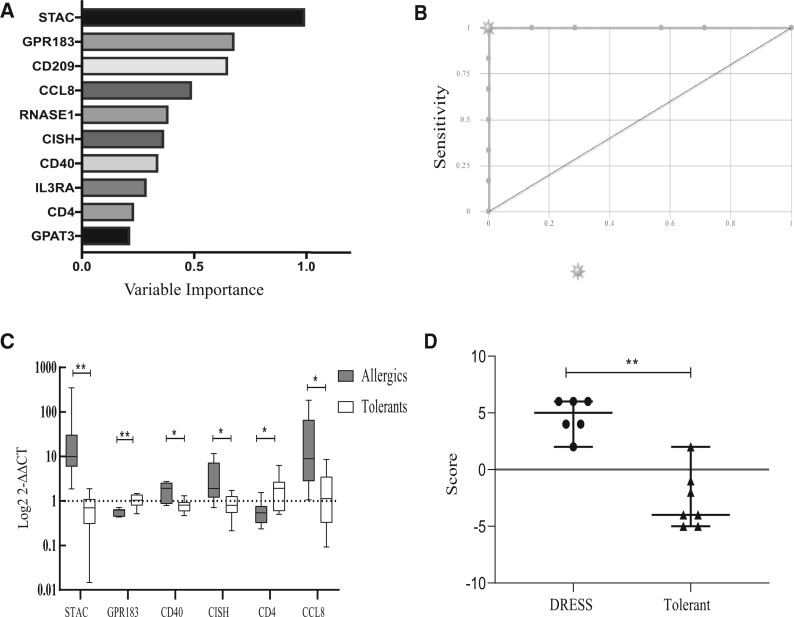
Optimization of biomarker panel to differentiate DRESS due to antibiotics from tolerant controls. A, Gene importance for the biomarker panel measured by random forest, 10 genes with the highest importance shown (Ranger package, R, α = 0.9, number of trees = 500, binary input). B, CombiROC analysis of 10 genes with highest importance (AUC = 1, sensitivity = 100%, specificity = 100%). C, Expression of genes in biomarker panel measured by qPCR in drug allergic patients (gray) and controls tolerant to specified antibiotics (white). Change induced in expression shown for 6 genes reaching statistical difference (* = *p* < .01) in expression change between patient cohorts (2^-ΔΔ^^*C*^^t^ versus YWHAZ housekeeping gene) plotted on a log10 scale. Box and whiskers indicate median and data range. D, Scatter plot of novel scoring system using 6 select biomarker genes to stratify DRESS and control subjects. Horizontal line indicates median score, error bars indicate data range. Horizontal red line indicates threshold score considered positive (**p < .01, Fisher’s exact test). Abbreviations: ROC, receiver operator characteristic; AUC, area under curve; CI, confidence interval. A color version of this figure appears in the online version of this article.

However, because it was apparent that not all genes contributed equally to the 22-gene scoring matrix that had been developed, we set out to evaluate which gene marker or combination of biomarkers had the highest predictive value for a prospective diagnostic test. Firstly, we took a machine learning approach and trained a random forest algorithm using the validation cohort data (Ranger package, R, α = 0.9, number of trees = 500, binary input). The analysis ranked the candidate biomarkers in order of importance for predictive classification (Figure 6A and Supplementary Table 3). For the 10 highest ranked markers, receiver operating characteristics (ROC) analysis showed 100% sensitivity and 100% specificity (AUC = 1). Secondly, we tested a reduced panel of biomarkers identified by their individually significant differential expression between allergics and tolerants: *STAC*, *GPR183*, *CD40*, *CISH*, *CD4*, and *CCL8* (Figure 6C) in contrast to the other genes in the 22-gene panel (Supplementary Figure 2). By applying our scoring algorithm manually to these 6 genes using a threshold score of 0, we enhanced the diagnostic accuracy as compared with the 22-panel (sensitivity 100%, specificity 85.7%; *p* = .0047, Fisher’s exact test; Figure 6D;Supplementary Table 4).

## DISCUSSION

Criteria for diagnosis of DRESS are clear: cutaneous eruption with hematologic abnormalities and systemic involvement, with the addition of HHV-6 reactivation by Japanese criteria (Shiohara *et al.*, 2007; Shiohara and Mizukawa, 2019). However, the optimal diagnostic work-up to identify a causal drug has remained elusive. Key to the management of DRESS is prompt discontinuation of the culprit drug, as the process can be progressive and even result in catastrophic organ failure (Bocquet et al., 1996; Kardaun *et al.*, 2013) and latterly autoimmune sequelae. The determination of drug culpability based only on chronological history of drug ingestion is often unreliable because of heterogeneous presentations and sometimes confusing long-latent periods following the introduction of drugs. In addition to this, definitive challenge testing is inadvisable in DRESS, leaving few alternative options for diagnostic assessment. Whilst some groups, including ourselves, have utilized *in vitro* functional T cell assays in an attempt to elucidate the causal drugs (Haw *et al.*, 2016; Mayorga *et al.*, 2016; Polak *et al.*, 2013), multiple issues restrict the widespread availability of such assays. These include the need for specialist resources and expertise, as well as variation in reported sensitivity of tests suggesting a user-dependent variability (Mayorga *et al.*, 2016, 2019). There is a clear need for new approaches to consideration of diagnostic testing modality for conditions such as DRESS.

By using a nonhypothesis driven approach to evaluate DRESS activated molecular pathways, we sought to maximize the possibility to detect a DRESS-specific signature. Furthermore, such an approach also contributes to better understanding of disease pathogenesis (Finotello *et al.*, 2019; Reuter *et al.*, 2015). Transcriptomic profiling by RNA sequencing is advantageous as not only does it enable identification of key DEGs but also has high sensitivity for low abundance transcripts (Jabbari *et al.*, 2012; Schwingen *et al.*, 2020). Utilization of RNAseq in melanoma (Berger *et al.*, 2010; Valsesia *et al.*, 2011), psoriasis and atopic dermatitis (Schwingen *et al.*, 2020) has enabled classification based on phenotype, prognosis, and prediction of intervention outcome. The availability of such technology should therefore be harnessed to further our understanding of cutaneous drug reactions to enable emergent clinical applications.

Here, using a multimethod, unbiased analysis approach, we identified 22 genes which were differentially regulated in blood cells from allergic individuals after *in vitro* exposure to the culprit drug. Of the 22 transcripts identified, we used a machine learning approach to select 10 and differential expression approach to select 6 with the strongest association with DRESS. *GPR183* (G-protein coupled receptor 183; syn. Epstein-Barr virus (EBV) -induced gene 2, *EBI2*) is expressed in lymphocytes where, by binding oxysterols, it creates a chemotactic gradient to direct movement of B cells, T cells, dendritic cells, and monocytes/macrophages (Benned-Jensen *et al.*, 2012; Hannedouche *et al.*, 2011). Downregulation of GPR183 as induced by exposure to the culprit drug in allergics in this study, has been shown to enhance production of type 1 IFNs and inflammatory cytokines by blood dendritic cells (Chiang *et al.*, 2013). Therefore, this may reflect an important pathway for enhanced drug-antigen presentation to CD8+ T cells in DRESS, which may contribute to the organ damage seen in this condition (Picard *et al.*, 2010). Viral reactivation, including human herpes virus 6 (HHV6), HHV7, EBV, and cytomegalovirus have been detected in cases of DRESS, postulated to be due to either direct drug or metabolite effect or alterations in immunity as result of antidrug response (Cho *et al.*, 2017). The exact role of viruses either as co-stimulating driver in disease onset or as a result of Treg dysregulation remains unclear but findings of alteration in type 1 IFN signaling would be in keeping with current understanding of viruses being interlinked with DRESS. CD4 downregulation is well established as a consequence of Th2 activation. The downregulation of CD4 expression in allergics following drug exposure as seen here is interesting because evidence of drug-specific HLA-restriction in DRESS has so far only identified MHC Class I alleles (Mullan *et al.*, 2019). These results therefore support the possibility that drug-specific CD4+ T cells may play an important role in DRESS. Further evidence of the role of CD4 activation is suggested by the enhanced CCL8 expression in allergics. CCL8 has been shown to be central to recruiting IL-5 producing Th2 cells (Islam *et al.*, 2011), which in turn regulate eosinophilia, thus linking these transcript changes to the hallmarks of DRESS. In addition, *CISH* (cytokine inducible SH2 containing protein), was found to be upregulated by culprit drug exposure in allergics and has been shown to be a marker of allergen-specific Th2 cells (Nakajima *et al.*, 2008), with a role in negative regulation of cytokines in the JAK-STAT5 pathway. Taken together, these data suggest an important role for drug-specific Th2 cells in DRESS and raise the possibility of therapeutic targeting of the Th2 pathway in acute disease. Recent drugs are already licensed for such purposes to treat other Th2 diseases including those targeting IL-4Ra, and anti-IL5. *STAC* (SH3 and cysteine-rich containing protein), a mediator of calcium-dependent inactivation, was also upregulated in DRESS and whilst it is likely to be important in regulating inflammation (Flucher and Campiglio, 2019), the precise role of *STAC1* (as here), remains to be established.

For diagnostic approaches, the sensitivity and specificity of the identified signature is key. Using a machine learning approach, we selected 10 genes which were demonstrated a sensitivity and specificity of 100%. However, to demonstrate conservative assessment of the utility of these biomarkers in DRESS, we showed that a combined panel of 6 genes, identified by differential gene expression statistics within the validation cohort allowed identification of the causative antibiotic in DRESS with greater accuracy than that of the initial 22 gene algorithm (sensitivity 100%, specificity 85.7%). These gene expression profiles were not evident in healthy volunteers who tolerated the drugs in question, and were not induced in other inflammatory conditions, which can mimic or precede onset of DRESS. This is an important consideration as multiple conditions can present similarly to DRESS.

Kim *et al.* recently applied single-cell RNA sequencing (scRNA-seq) to a single case of sulfamethoxazole/trimethoprim DRESS, and identified transcriptomal alterations in associated with proliferation, migration, activation and signaling pathways, which then informed therapeutic options (Kim *et al.*, 2020). Whilst such an approach may be ideal, scRNA-seq applicability to clinical practice is limited by high cost and need for expertise. A wholly *ex vivo* diagnostic test is safe and requires only a minimal amount of blood sampling from patients. Optimization of a test based on PBMCs mitigates the need for cell sorting which would limit feasibility for widespread use. Gene signatures derived would be inclusive of T cell activation amongst other components of PBMCs, an important consideration in DRESS. Moreover, as the incidence of DRESS is relatively low, between 1:1000 to 1:10 000 drug exposures (Fiszenson-Albala *et al.*, 2003), our preferred approach is to utilize a paired analysis (control vs drug) in diagnostic samples, which mitigates the need for validation of normal ranges for population-wide background correction. Of note, the exact genes involved in the JAK-STAT pathways in this publication were not significantly differentially expressed in our cohorts, potentially reflecting differences in active DRESS state as compared with following recovery or differences in drug effects. Further elucidation of the utility of the potential gene panel we have identified in other diseases states, that is, acute or on-going DRESS and with other medications will be necessary.

The limitations of this work include the sample size, and the restriction of the allergic cohorts tested to antibiotic induced DRESS. Due to DRESS being a relatively uncommon condition (Fiszenson-Albala *et al.*, 2003), subject numbers with a single definite causative drug is limited. As significant heterogeneity exists amongst affected subjects and there are likely pathomechanistic variations of differing drugs, for this pilot study, we limited inclusion to a single class of medications, that is, antibiotics as these were the subjects most frequently referred to our center for diagnostic investigations. It remains uncertain whether this transcriptomic signature can be applied to other larger cohorts of DRESS subjects due to other medication and different phenotypes, for example, Stevens-Johnson syndrome. Future work involving comparison of current biomarker panel with DRESS induced by other classes of medications as well as in larger cohorts will be crucial. This will entail a considerable duration give the relatively low incidence of DRESS. Additionally, our tested patients with DRESS were otherwise well at the time of sampling, and therefore, we have no data on the utility of this test in acutely ill patients. Whilst testing during the recovery phase enables baseline drug-induced activation to be established, it is likely there are multiple components to DRESS signature changes with differences between the acute and resolution phase or even variability dependent on the DRESS-phenotype. Gene expression profiles identified in this study are not specific to T cells due to usage of PBMCs and would have included other cellular components. Whilst comparison had been performed on whole blood transcriptome in 3 comparator datasets due to absence of RNAseq data from PBMCs only, pipeline processing would not have significantly differed and observed alterations would have been inclusive of those in PBMCs. To compensate for the possible differences in sample composition, comparison was carried out for specific gene signatures, independent of other genes expressed in comparator samples.

In summary, we have identified a potential panel of gene transcripts, which can be measured on a preprinted array card, which may offer a useful diagnostic test in antibiotic-associated DRESS with a conservative assessment of 85.7% prediction rate (0.48%–0.99 95% CI), and sensitivity of 100% and specificity of 85.7%. The advantage of this approach is that such gene card testing is familiar to hospital laboratories and therefore this technology is scalable for routine use. Further work is required to determine whether the same panel can be used for larger cohorts, different medications, and other T–cell-mediated drug hypersensitivity reactions.

## AUTHOR CONTRIBUTIONS

Y.X.T. and W.Y.H. performed experiments, analyzed data and drafted the manuscript. A.F.V. analyzed data and provided technical support. C.G. performed experiments. J.W. analyzed data. P.S.F. contributed to study design and oversaw writing of the manuscript. M.E.P. and M.A.J. designed the research study, analyzed data and oversaw writing of the manuscript.

## SUPPLEMENTARY DATA

Supplementary data are available at *Toxicological Sciences* online.

## Supplementary Material

kfac062_Supplementary_Data

## Notes

Marta Ewa Polak and Michael Roger Ardern-Jones share senior authorship.

The authors certify that all research involving human subjects was done under full compliance with all government policies and the Helsinki Declaration.
