# Supplementary material for: Potential Biomarker Identification by RNA-Seq Analysis in Antibiotic-Related Drug Reaction with Eosinophilia and Systemic Symptoms (DRESS): A Pilot Study
Source: Toxicol Sci. 2022 Jun 15;189(1):20–31. doi: 10.1093/toxsci/kfac062 (PMC9412178; doi:10.1093/toxsci/kfac062)
Supplement: kfac062_Supplementary_Data [file kfac062_supplementary_data.docx]

Supplementary Material

**
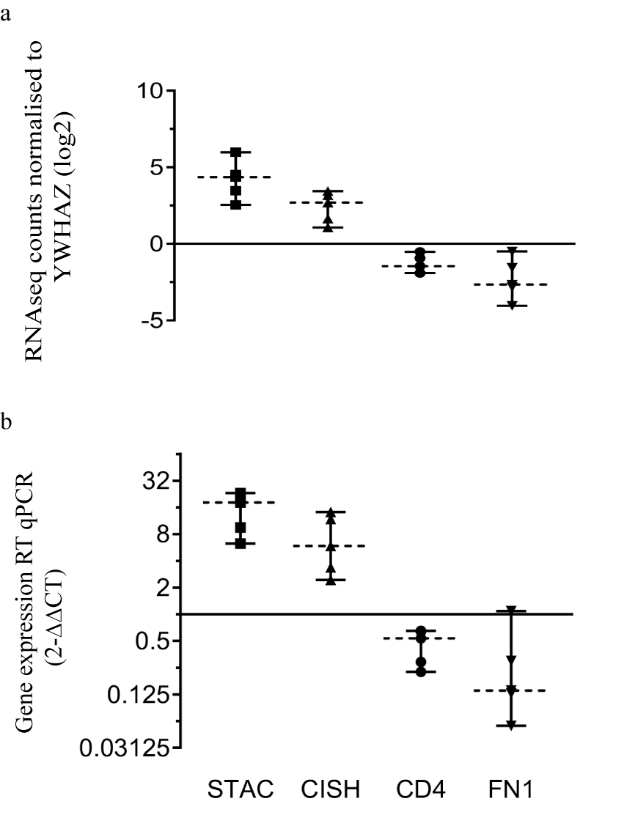
Supplemental Figure 1**

**Supplemental Figure 1.** Validation of top DEGs identified by whole transcriptome analysis. Changes in gene expression detected by RNA-sequencing were reproduced using RT-qPCR for the top 4 gene transcripts. a) Gene expression measured using RNA-sequencing for *STAC*, *CISH*, *CD4* and *FN1*. Log_2_ of TMM normalised counts calibrated to *YWHAZ* b) Gene expression measured using RT-qPCR for *STAC*, *CISH*, *CD4* and *FN1* 2^-ΔΔCT^, *YWHAZ* as housekeeping gene, in duplicate. Each data point represents gene expression in a single patient. Horizontal dotted line shows group median, error bars depict data range.


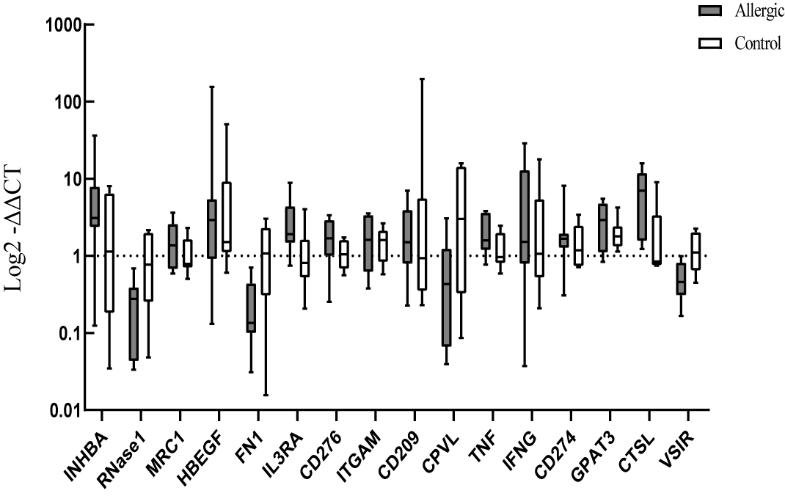
**Supplemental Figure 2**

**Supplemental Figure 2.** Expression of remaining genes from gene panel in validation cohort and in tolerant subjects. Expression of genes in biomarker panel measured by qPCR in drug allergic patients (grey) and controls tolerant to specified antibiotics (white). Change induced in expression shown for genes not reaching statistical difference of p = <0.05 in expression change between patient cohorts (2^-ΔΔCT^ versus *YWHAZ* housekeeping gene). Box and whiskers indicate median and data range.


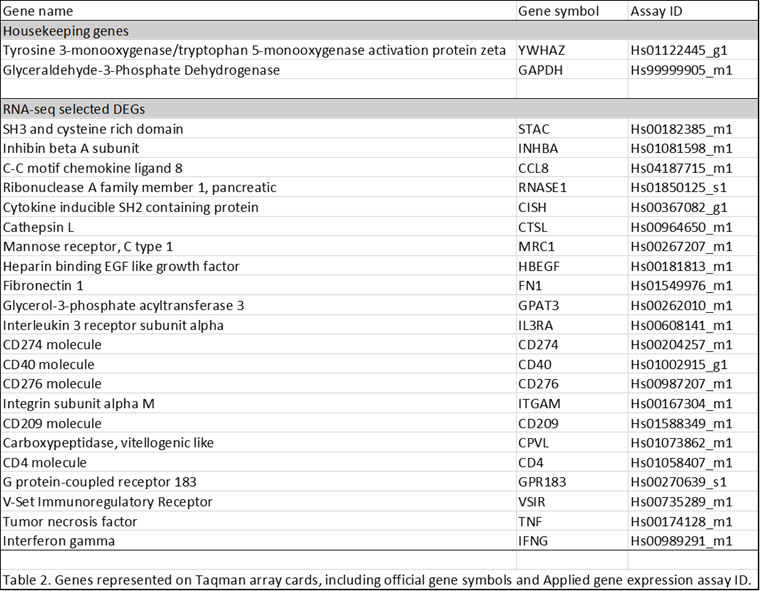
**Table S1:** Genes included in biomarker panel.

**Table S2:** Scoring using 22 biomarker genes in identification of DRESS subjects

| **Biomarker** | DRESS 1 | DRESS 2 | DRESS 3 | DRESS 4 | DRESS 5 | DRESS 6 | Tolerant 1 | Tolerant 2 | Tolerant 3 | Tolerant 4 | Tolerant 5 | Tolerant 6 | Tolerant 7 |
| --- | --- | --- | --- | --- | --- | --- | --- | --- | --- | --- | --- | --- | --- |
| CTSL | 1 | 1 | 1 | 1 | 1 | 1 | 0 | 1 | -1 | -1 | -1 | -1 | 1 |
| IL3RA | 1 | 1 | 1 | 1 | 1 | -1 | -1 | 0 | 0 | -1 | -1 | -1 | 1 |
| CISH | 1 | 1 | -1 | 1 | 1 | 1 | -1 | 1 | 0 | -1 | -1 | -1 | -1 |
| TNF | 0 | 1 | 1 | 1 | 1 | -1 | 0 | 1 | -1 | 0 | 0 | -1 | 1 |
| ITGAM | 1 | 1 | -1 | 0 | 1 | -1 | -1 | 1 | 1 | 1 | 1 | -1 | 1 |
| HBEGF | 0 | 1 | 1 | 1 | 1 | -1 | 1 | 1 | 0 | 1 | 1 | -1 | 0 |
| CD276 | 1 | 0 | 1 | 1 | 1 | -1 | -1 | 0 | -1 | 1 | 1 | 0 | 0 |
| INHBA | 1 | 1 | 1 | 1 | 1 | -1 | 1 | 1 | 0 | -1 | -1 | -1 | 0 |
| GPAT3 | 1 | 0 | 1 | 1 | 1 | 0 | 0 | 1 | 1 | 1 | 1 | 1 | 1 |
| CCL8 | 1 | 1 | 1 | 1 | 1 | 1 | 1 | 1 | -1 | -1 | -1 | 0 | 1 |
| CD274 | 1 | 1 | -1 | 1 | 1 | 1 | 1 | 1 | 0 | -1 | -1 | -1 | 1 |
| CD209 | -1 | 1 | 0 | -1 | 1 | 1 | 1 | -1 | -1 | 0 | 0 | 1 | -1 |
| CD40 | 0 | 1 | -1 | 1 | 1 | 1 | -1 | 0 | -1 | -1 | -1 | 0 | 1 |
| MRC1 | -1 | 1 | -1 | -1 | 1 | 1 | 1 | 1 | 1 | -1 | -1 | -1 | -1 |
| STAC | 0 | 1 | 1 | 1 | 1 | 1 | -1 | -1 | 0 | 0 | 0 | -1 | -1 |
| IFNG | 1 | 1 | -1 | 1 | 1 | -1 | -1 | 1 | -1 | -1 | -1 | 1 | 1 |
| GPR183 | 1 | 1 | 1 | 1 | 1 | 1 | -1 | -1 | -1 | -1 | -1 | -1 | 1 |
| RNASE1 | 1 | 1 | 1 | 1 | 1 | 1 | -1 | -1 | 1 | -1 | -1 | 1 | 1 |
| FN1 | 1 | 1 | 1 | 1 | 1 | 1 | -1 | -1 | 1 | -1 | -1 | -1 | 1 |
| CPVL | 1 | -1 | -1 | 1 | 1 | -1 | -1 | 1 | -1 | -1 | -1 | 1 | -1 |
| CD4 | 1 | -1 | 1 | 1 | 1 | 1 | -1 | -1 | -1 | -1 | -1 | 1 | 1 |
| VSIR | 1 | 1 | 1 | 1 | -1 | 1 | -1 | -1 | 1 | -1 | -1 | -1 | 1 |
| **Score** | **14** | **16** | **7** | **17** | **20** | **5** | **-7** | **5** | **-4** | **-11** | **-11** | **-7** | **9** |

**Table S2**. Scoring algorithm enabling differentiation of DRESS subjects from tolerant controls. A point was added if transcript expression matched expected up- or down-regulation of gene panel identified from the ‘discovery’ cohort whilst 1 point was subtracted if direction of change was opposite to that of the identified signature. No points were allocated or subtracted if log_2_ fold change values fell between -0.25 and 0.25. Threshold score of 6 scored against all 22 genes showed sensitivity of 83.3% and specificity of 85.7% (6 DRESS, 7 tolerant controls; p=0.029, Fisher’s exact test).

**Table S3:** Biomarker genes ranked according to random forest algorithm values.

**Table S4:** Scoring using 6 selected biomarker genes in identification of DRESS subjects.

| **Biomarker** | DRESS 1 | DRESS 2 | DRESS 3 | DRESS 4 | DRESS 5 | DRESS 6 | Tolerant 1 | Tolerant 2 | Tolerant 3 | Tolerant 4 | Tolerant 5 | Tolerant 6 | Tolerant 7 |
| --- | --- | --- | --- | --- | --- | --- | --- | --- | --- | --- | --- | --- | --- |
| CISH | 1 | 1 | -1 | 1 | 1 | 1 | -1 | 1 | 0 | -1 | -1 | -1 | -1 |
| CCL8 | 1 | 1 | 1 | 1 | 1 | 1 | 1 | 1 | -1 | -1 | -1 | 0 | 1 |
| CD40 | 0 | 1 | -1 | 1 | 1 | 1 | -1 | 0 | -1 | -1 | -1 | 0 | 1 |
| STAC | 0 | 1 | 1 | 1 | 1 | 1 | -1 | -1 | 0 | 0 | 0 | -1 | -1 |
| GPR183 | 1 | 1 | 1 | 1 | 1 | 1 | -1 | -1 | -1 | -1 | -1 | -1 | 1 |
| CD4 | 1 | -1 | 1 | 1 | 1 | 1 | -1 | -1 | -1 | -1 | -1 | 1 | 1 |
| **Score** | **4** | **4** | **2** | **6** | **6** | **6** | **-4** | **-1** | **-4** | **-5** | **-5** | **-2** | **2** |

**Table S4**. Modification of scoring algorithm with inclusion of select 6 genes. A point was added if transcript expression matched expected up- or down-regulation of gene panel identified from the ‘discovery’ cohort whilst 1 point was subtracted if direction of change was opposite to that of the identified signature. No points were allocated or subtracted if log_2_ fold change values fell between -0.25 and 0.25. Scoring to 6 identified genes with threshold score of 0 showed improved sensitivity and specificity (sensitivity 100%, specificity 85.7%; p = 0.0047, Fisher’s exact test).

**Table S5:** Inflammatory diseases gene expression signature overlap with biomarker panel analysis.

| **Disease** | **Curated gene set**  **(GEO dataset)** | **Gene expression in curated gene set** | **Number of genes** | **Normalised enrichment score** | **FDR q-value** | **Overlap genes with DRESS biomarker** |
| --- | --- | --- | --- | --- | --- | --- |
| Influenza | GSE6269: healthy vs influenza PBMC | Upregulated | 160 | 1.458515 | 0.006908463 |  |
| Sepsis | GSE9960: healthy vs sepsis PBMC | Upregulated | 192 | 2.433338 | 0.0 | STAC |
|  | GSE9960 healthy vs gram positive sepsis PBMC | Upregulated | 198 | 2.1894507 | 0.0 | INHBA, CD274 |
|  | GSE9960 healthy vs gram negative sepsis PBMC | Downregulated | 198 | 2.4648156 | 0.0 | INHBA |
|  | GSE9960 healthy vs gram negative and positive sepsis PBMC | Downregulated | 196 | 1.4939677 | 0.004305705 | CCL8 |
| Systemic lupus erythematosus | GSE10325 myeloid vs lupus | Upregulated | 190 | 2.4966972 | 0.0 | CCL8 |
|  | GSE10325 CD4 T cell vs lupus CD4 T cells | Upregulated | 157 | 2.166506 | 0.0 |  |
|  | Bennett systemic lupus erythematosus M12175 | Upregulated | 27 | 1.3876796 | 0.04183008 |  |
|  | GSE30153 lupus vs healthy donor B cells | Upregulated | 146 | 1.1878923 | 0.1454361 |  |
|  | GSE10325 myeloid vs lupus myeloid | Downregulated | 70 | 1.2539403 | 0.10766052 |  |
| Dermatomyositis | Disgenet dermatomyositis CUI: C0011633 | Upregulated | 149 | 1.5843996 | 0.0034364262 | TNF, CD274, CTSL, MRC1 |

DRESS = drug reaction with eosinophilia and systemic symptoms; FDR = false discovery rate; GEO = gene expression omnibus; PBMC = peripheral blood mononuclear cells; vs = versus
